# “It spreads like wildfire”: mothers’ gatherings for vaccine acceptance

**DOI:** 10.3389/fpubh.2024.1198108

**Published:** 2024-08-27

**Authors:** Diego de Acosta, Temple Moore, Fariha Alam, Sarah J. Hoffman, Megan Keaveney, Erin Mann, Elizabeth Dawson-Hahn

**Affiliations:** ^1^National Resource Center for Refugees, Immigrants, and Migrants, University of Minnesota, Minneapolis, MN, United States; ^2^Refugee Women’s Network, Decatur, GA, United States; ^3^School of Nursing, University of Minnesota, Minneapolis, MN, United States; ^4^Centers for Disease Control and Prevention, Division of Global Migration and Quarantine, Immigrant, Refugee and Migrant Health Branch, Atlanta, GA, United States; ^5^Department of Pediatrics, University of Washington, Seattle, WA, United States

**Keywords:** community health workers (CHWs), human-centered design (HCD), COVID-19, peer support (PS), refugees and immigrants, mothers, COVID-19 vaccine acceptance, family health

## Abstract

This case study describes the design, implementation, and evaluation of an initiative to increase COVID-19 vaccine confidence and uptake among refugee and immigrant women in Clarkston, Georgia. Applying the principles and practices of human-centered design, *Mothers x Mothers* was co-created by Refugee Women’s Network and IDEO.org as a series of gatherings for refugee and immigrant mothers to discuss health issues, beginning with the COVID-19 vaccine. The gatherings included both vaccinated and unvaccinated mothers and used a peer support model, with facilitation focused on creating a trusting environment and supporting mothers to make their own health decisions. The facilitators for *Mothers x Mothers* gatherings were community health workers (CHWs) recruited and trained by Refugee Women’s Network. Notably, these CHWs were active in every phase of the initiative, from design to implementation to evaluation, and the CHWs’ professional development was specifically included among the initiative’s goals. These elements and others contributed to an effective public health intervention for community members who, for a variety of reasons, did not get sufficient or appropriate COVID-19 vaccine information through other channels. Over the course of 8 *Mothers x Mothers* gatherings with 7 distinct linguistic/ethnic groups, 75% of the unvaccinated participants decided to get the COVID-19 vaccine and secured a vaccine referral.

## 1 Introduction

Community health workers (CHW) facilitate connections between health systems and communities, providing vital outreach and education to underserved populations (1, 2). CHW programs, once primarily associated with low-resource settings in the Global South, have more recently attracted interest in higher-resource settings, where health disparities persist despite well-funded and well-developed health systems (3, 4). The COVID-19 pandemic accentuated these disparities globally and highlighted the importance of community-level health work, not only for the pandemic response but also for routine healthcare delivery (5, 6).

In the United States, CHWs’ roles and work settings are varied, but studies have shown that they create unprecedented levels of engagement in traditionally underserved communities (1, 4, 7). CHWs in the United States have facilitated innovative approaches to the primary, secondary, and tertiary prevention and management of common chronic diseases like diabetes, cardiovascular disease, HIV, and some cancers (4). During the COVID-19 pandemic, CHWs’ work in the United States included public health communication and education, health system navigation, occasional contact tracing and monitoring, and attending to the larger social, economic, and behavioral health needs of underserved communities (1, 7).

The CHW model of care figures prominently in a landmark 2020 paper by Holeman and Kane about the promise of human-centered design for global health equity (8). *Human-centered design* is an approach to systems-level problem-solving that prioritizes intended users’ perspectives and needs throughout an iterative design process (9). Holeman and Kane draw a parallel between the designer’s obligation to communities and the CHW’s responsibility to patients—“not only to deliver efficient biomedical services, but to accompany patients, to ease suffering and to offer their caring presence as an antidote to despair” (8). By imagining health-equity-oriented design as “accompanying a community,” Holeman and Kane emphasize the need to cultivate relationships with community members, understand their experiences and perspectives, and build interventions with them (8).

## 2 Background and rationale

CHWs have historically served as community health liaisons, but there was an additional need during the COVID-19 pandemic for CHWs to provide critical services to refugee and immigrant communities, like providing support for case investigation and contact tracing (CICT), facilitating access to vaccines, and conducting education and outreach (7, 10, 11). With regard to CICT and vaccine access, refugee and immigrant communities faced overlapping challenges due to different cultural practices around health and healthcare, difficulties accessing health information and services, community concerns about vaccines and, sometimes, distrust of government or health systems (12, 13). As for education and outreach, the COVID-19 pandemic challenged public health departments across the United States to develop clear messaging for diverse populations. Unfortunately, refugees and immigrants who navigate their social environments and media in languages other than English often missed out on crucial COVID-19 information due to messaging that was: (1) not adequately or completely translated, (2) not culturally or situationally concordant, or (3) not delivered through effective or accessible communication channels (14, 15).

Community-based organizations and CHWs, often working together, addressed some of these gaps and therefore served as crucial partners to public health departments in their efforts to connect refugees and immigrants to COVID-19 information and vaccines (7, 16). Community-based organizations adapted the COVID-19 public health response for local refugee and immigrant communities by providing culturally responsive education, services, and advocacy in ways that emphasized community strengths over mere compliance (11). Meanwhile, CHWs extended the reach of public health and health systems—as well as social service agencies—through culturally responsive communication and robust relationships built on trust, support, and advocacy (1, 17).

## 3 Context

Refugee Women’s Network (RWN) is a 501c3 non-profit in Northeastern Atlanta, Georgia, led by refugee and immigrant women since its founding 25 years ago. RWN serves refugee and immigrant families who have resettled in the state of Georgia, with a mission “to support women survivors of war, conflict, and displacement in overcoming cultural and systemic barriers to achieving healthy, self-sufficient, and fulfilling lives” (18). RWN’s work is largely based in Clarkston, a small city adjacent to the Atlanta metropolitan area that receives many of Georgia’s resettled refugees.

Of Clarkston’s 14,538 residents, 52.5% are foreign-born and 61.9% of those aged 5 or older speak a language other than English at home (19)–much higher than the corresponding national figures of 13.6 and 21.6%, respectively (20). These populations face challenges to getting relevant health information and services in their own languages from sources they trust (21).

In 2019 and 2020, Georgia State University Prevention Research Center conducted two multilingual surveys among Clarkston residents 18 and older who self-identified as refugees or asylees. Of those surveyed, 57% self-reported “marginal reading, writing and speaking skills in English” (21), significantly more than the 8.3% of U.S. residents who say they speak English less than “very well” (20). Some 31% of Clarkston survey respondents were living in high-density households (≥6 people) (21), while the average U.S. household has 2.54 people (20). Finally, among survey respondents, 66% reported high levels of daily stress, 82% had high levels of financial insecurity, and 70% “did not know where to get benefits like unemployment or financial assistance” (21) (Table 1).

**Table 1:** Case study description and methods.

This community case study describes a program implementation and evaluation conducted by Refugee Women’s Network (RWN), a community organization. Data were collected by RWN for programmatic purposes and are shared here in aggregate. This activity was reviewed by the CDC and was conducted consistent with applicable federal law and CDC policy. The case study is based on documents created by RWN and IDEO.org, as well as interviews with RWN and IDEO.org members. All materials were carefully reviewed to identify promising and replicable practices using criteria outlined by Ng and De Colombani (37). **Documents** RWN documents included four quarterly reports (2021–22) describing RWN’s NACCHO-funded work; the cumulative report for RWN’s NACCHO-funded work; RWN’s 2022 Community Health Promotion impact report; and a data tracking spreadsheet for *Mothers x Mothers.* IDEO.org documents included two slide decks outlining the design process for *Mothers x Mothers.* **Interviews** Interviews with RWN and IDEO.org served to further contextualize the design and implementation of *Mothers x Mothers*, as well as to integrate the perspectives of key players. There were 4 semi-structured interviews: 3 interviews with RWN’s Community Health Promoters and 1 with the Program Manager for Community Health Promotion. There was 1 further in-depth interview with the Program Manager for Community Health Promotion. There was 1 in-depth interview with designers from IDEO.org who worked on *Mothers x Mothers*. Interviews were conducted via Zoom between 5/9/2022 and 12/7/2022, audio-recorded and professionally transcribed. Interview transcripts were used to identify key excerpts and confirm the accuracy of quoted material.

## 4 *Mothers x mothers*: key programmatic elements

In November 2021, RWN launched an innovative community health initiative called *Mothers x Mothers*. This initiative was made possible by a grant from the National Association of City and County Health Officials (NACCHO), which funded local health departments and community-based organizations around the United States to promote COVID-19 mitigation and management strategies in refugee and immigrant communities (22). The *Mothers x Mothers* initiative was also supported by the National Resource Center for Refugees, Immigrants, and Migrants (NRC-RIM) (23).

The *Mothers x Mothers* initiative had three goals: (1) to increase vaccine confidence among refugee and immigrant mothers, (2) to empower these same mothers to make informed decisions about their own health more generally, and (3) to gain enough community trust to sustain future interventions. *Mothers x Mothers* was jointly designed by RWN and IDEO.org, an international 501c3 nonprofit specializing in human-centered design for health equity, community engagement, and humanitarian aid.

### 4.1 Community health gatherings

*Mothers x Mothers* was a series of gatherings designed for participants with shared identities, experiences, and interests. The gatherings used a peer support model and were structured around a template that included icebreaker questions, safe space agreements, and a Google Slides deck about selected health topics, starting with COVID-19 and the COVID-19 vaccine. Peer support has been used as an intervention model to address issues ranging from mental health (24) to perinatal maternal health (25) to breastfeeding (26) to vaccine hesitancy and misinformation (27). Peer support appears to be most effective when organizations co-design culturally concordant programs with communities rather than imposing ideas top-down (28).

Each group that gathered for *Mothers x Mothers* at RWN consisted of refugee and immigrant mothers who were living in Clarkston and shared a language or region of origin. There were seven groups in all: one each for Burmese-, Arabic-, Sango-, French-, Swahili-, Somali-, and Amharic- and Tigrinya-speaking mothers (the last two being combined into a single group). Each gathering was facilitated by a *Community Health Promoter* (RWN’s job title for a CHW). The first 1–2 gatherings for each group focused on the COVID-19 vaccine, and the slides for these meetings presented facts to counter misinformation about COVID-19 and the vaccine common to each community. Through deliberate recruitment, some of the participants in these gatherings were unvaccinated while others had already received the COVID-19 vaccine. As one Community Health Promoter explained,

“You’re more likely to believe someone who is in a similar life situation as you, and if a unvaccinated mother hears from a vaccinated mother who’s the exact same as her, has kids in the same age range, speaks the same language, they’re more likely to change their mind.”

After the first 1–2 gatherings focused on COVID-19, the Community Health Promoters asked group members to choose the future direction of the gatherings. The manager of the program stated:

“[We] honor them, saying, ‘Okay, we pushed COVID the first two meetings, but now it’s yours,’ like we want this to be a sustainable model so that these women are looking forward to coming back every month, and we want them to drive it.”

Examples of topics chosen by participants were nutrition and diet, high blood pressure, diabetes, and oral hygiene.

### 4.2 Human-centered design involving community representatives

**Figure 1:**
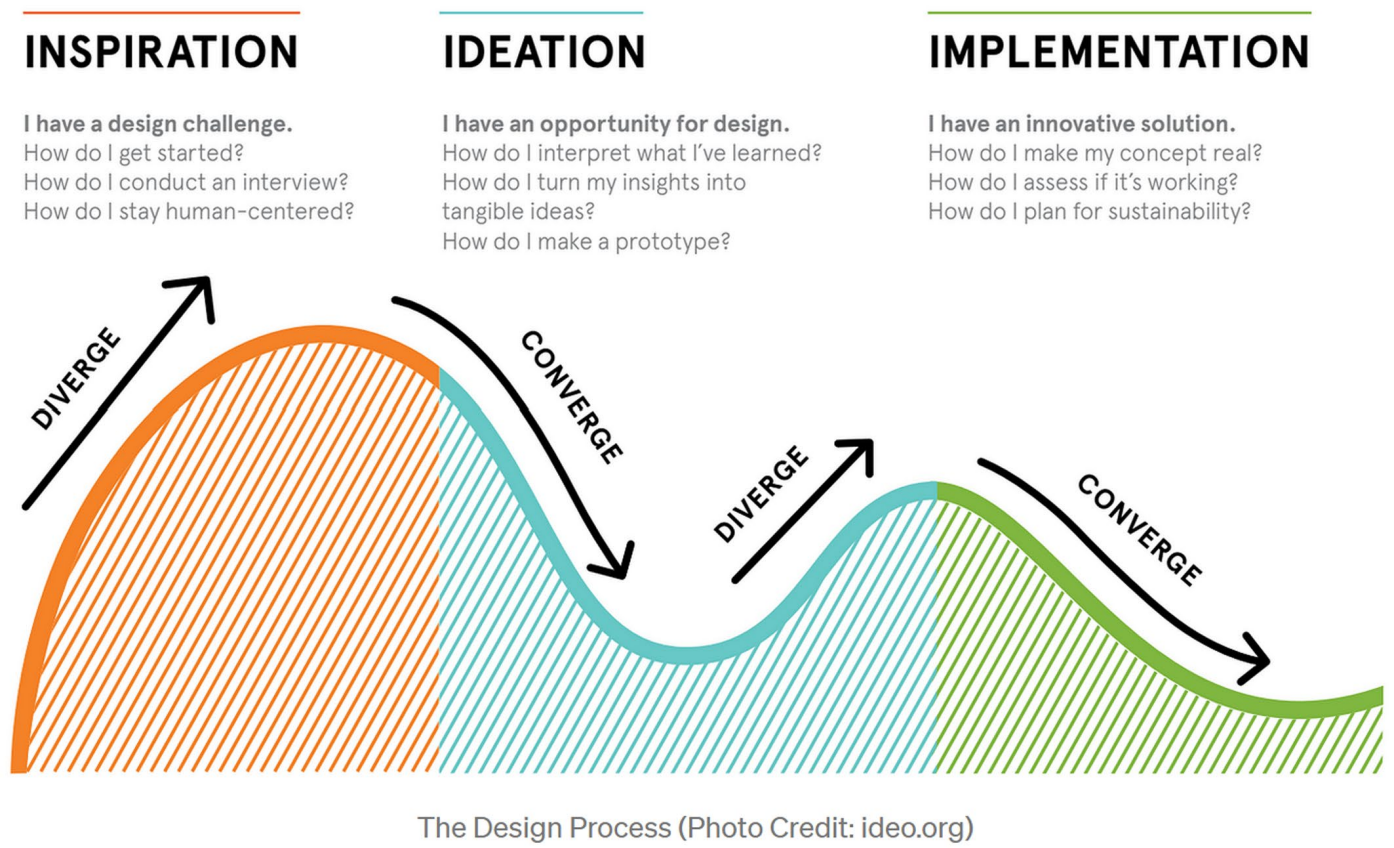
The human-centered design process.

A particularly innovative aspect of *Mothers x Mothers* was the design process that paired IDEO.org designers with a group of women co-designers from the Somali, Eritrean, and Ethiopian communities, including RWN Community Health Promoters. Using a human-centered design process (Figure 1)—i.e., one that actively involved the intended users throughout the iterative design process—the diverse team worked to understand the specific challenges facing refugee and immigrant women during the COVID-19 pandemic (inspiration), creatively explored a range of possible culturally-concordant solutions (ideation), and developed prototypes to test the viability of specific interventions (implementation).

Early in this process, IDEO.org and RWN considered a wide range of ideas to address vaccine hesitancy, from relatively modest proposals (e.g., peer-to-peer conversations) to technologically sophisticated ones (e.g., automated messaging via WhatsApp). When IDEO.org designers found the idea of peer support gaining traction, they proceeded with the broad idea of gatherings of mothers using a peer support model. Why peer support? Co-designers from the community had noted that vaccination was not common in their countries of origin, making the support of like-minded peers crucial for mothers considering the COVID-19 vaccine (29). Additionally, during the inspiration phase, co-designers had shared anecdotes that demonstrated the impact of social endorsements–negative as well as positive–regarding the vaccine.

At this point, more options were weighed and discussed: in-person vs. virtual gatherings; gatherings primarily focused on COVID-19 vs. gatherings coupling COVID-19 with a low-stakes theme like food; gatherings pairing mothers with Community Health Promoters, religious leaders, friends, or their own children. Design team members were most enthusiastic about in-person gatherings for mothers, facilitated by a Community Health Promoter. One of the aims of these gatherings would be to create space for mothers to hear positive social endorsements of the COVID-19 vaccine.

The next step in the design process was to start holding in-person prototype gatherings, carefully observing and assessing how the sessions went, and making needed refinements to the concept and organization of gatherings. Two prototype gatherings were held, one with Somali mothers and another with Eritrean and Ethiopian mothers. In this phase, all 13 participating mothers were unvaccinated, and 4 decided to get the vaccine as a result of the prototype gatherings.

Later, as recruitment for pilot gatherings and regular *Mothers x Mothers* gatherings ramped up, RWN began to include vaccinated mothers alongside unvaccinated ones. At first, this was an accident of the recruitment process: RWN did not ask participants for their vaccination status before gatherings so that they would not feel singled out or discouraged from joining just because they did not hold a specific view about vaccination. However, the mix turned out to be fortuitous: in a mixed gathering, unvaccinated mothers could learn from vaccinated mothers about their views and experiences, while vaccinated mothers had an opportunity to share their experiences and also raise concerns about vaccinating their children. Realizing the potential for greater impact, RWN made a special effort to recruit both vaccinated and unvaccinated mothers from that point forward.

### 4.3 Roles

#### 4.3.1 Community health promoters

Much of CHWs’ work falls under the rubric of health promotion, that is, encouraging health awareness and specific health practices through education and counseling (2). Starting in late 2019, RWN began recruiting and hiring CHWs, called *Community Health Promoters*, from the refugee and immigrant communities the organization serves. Twenty of these Community Health Promoters have been recruited, hired, and trained since then. Collectively, they speak 18 languages: Amharic, Arabic, Bengali, Burmese, Chin (Hakha), Dari, English, French, Hindi, Kurdish, Lingala, Pashto, Rohingya, Somali, Sango, Spanish, Swahili, and Tigrinya.

Each Community Health Promoter received 15+ hours of required standard training, in addition to ongoing monthly opportunities for further optional training on emerging topics of interest. The required training included modules on health communication, motivational interviewing, COVID-19 and COVID-19 vaccines, vaccine education, health insurance basics, and healthcare navigation for new refugees, in addition to basics about chronic disease, nutrition, and women’s health. The Community Health Promoters were trained to facilitate group conversations, refer community members to health services, help community members navigate vaccine clinics, and more.

In the design of *Mothers x Mothers,* the Community Health Promoters facilitated gatherings, created a trusting environment, and supported mothers to make health decisions that were in their own best interests. They were not expected to act as experts on COVID-19 or the COVID-19 vaccine; however, they knew where to refer mothers for COVID-19 information. The Community Health Promoters also worked to increase access to services by providing information about testing and vaccination locations, as well as guidelines for staying safe and keeping others safe. As one Community Health Promoter pointed out, the key to doing this work effectively was

“a lot of communication training, like how do you talk to people without bias, being neutral and third person… ways to back off in an argument that might be happening… because people do get angry and people will argue with you about COVID, so when is the right time to back off? When is the right time to respect someone’s boundary and just let the situation go?”

Thus it was especially important for the Community Health Promoters to build and refine group communication skills.

#### 4.3.2 Mothers

The *Mothers x Mothers* initiative specifically focused on mothers, who RWN recognizes as “health broker[s] of the family and community” (18). In an early phase of design, IDEO.org had worked toward ways to increase vaccine uptake among refugee and immigrant adolescents, but found that “designing with and for their mothers was crucial as they are the advocates—and gatekeepers—of their children’s health” (29). This realization aligns with research showing that in the United States, it is mothers who make about 80% of healthcare decisions for their children (30). Moreover, while there are many cultural and situational factors in play, in many cases it is mothers, not fathers, whose level of vaccine confidence predicts whether children will be vaccinated (31, 32). In the United States, mothers have generally been more hesitant than fathers to accept the COVID-19 vaccine for their children (33, 34), making it vitally important to reach mothers and address their concerns.

#### 4.3.3 Content experts

Content experts participated in the *Mothers x Mothers* program by contributing ideas for slides and sometimes sharing information in person, but their role was intentionally limited to give priority to peer support. As the manager of the program explained, “we try to make everyone feel on the same level, so a Health Promoter is there, but the Health Promoter, by nature, is on the same level.” At one point, one of the groups asked for a doctor to be present at their next gathering. RWN acknowledged the request and arranged for a culturally and linguistically concordant doctor to be present, but structured the meeting so that there was no expectation that the doctor would give a lecture or be positioned “above” the group of mothers in some other way.

Reflecting on how well peer support had worked to shift vaccine confidence in *Mothers x Mothers* groups, the program manager said,

“It wasn't because it was a… doctor, it was because there was a bunch of women from those communities that related to each other, and we created a safe atmosphere where they could share their concerns and they could have dialogue… especially because that social network was so damaged during COVID.”

### 4.4 Logistical coordination

RWN identified ways to expand opportunities for engagement, not only for mothers to get COVID-19 tests and vaccines, but also for them to participate in the kind of open peer-to-peer conversation about COVID-19 vaccines that *Mothers x Mothers* provided. They offered three kinds of help to *Mothers x Mothers* participants:

1. Transportation in the form of rides provided by volunteers, organized carpooling, or reimbursement for mileage;
2. Childcare provided on-site during gatherings so the mothers could take a break from caregiving, deeply engage in the activity, and connect with other women from their community; and,
3. Other incentives, such as gift cards, diapers, hygiene products, or food boxes, to compensate for the time and effort it took to come to the gatherings.

RWN’s Community Health Promoters recruited participants for *Mothers x Mothers* by distributing flyers and talking face-to-face with potential attendees. The Community Health Promoters emphasized that the gatherings would be welcoming, non-judgmental spaces, and mentioned the help and incentives described above.

## 5 Evaluation: process and outcomes

RWN’s leadership and Community Health Promoters placed equal emphasis on process and outcome evaluation. All case study interviewees spoke of wanting an initiative that prioritized trust, sustainability, and participant-led programming, with an accompanying benefit of increasing vaccine uptake. They conceptualized and operationalized *Mothers x Mothers* through three process-oriented goals: (1) building vaccine confidence, (2) building trust, and (3) building capacity.

### 5.1 Building vaccine confidence

RWN built vaccine confidence by promoting honest, informed discussion about COVID-19 so that community members could consider vaccination and get vaccinated, especially in more vaccine-hesitant communities. Table 2 shows the shift in vaccination status among *Mothers x Mothers* participants. Over the course of 8 *Mothers x Mothers* gatherings with 7 distinct groups, there were 49 participants (36 vaccinated, 1 unknown, 12 unvaccinated). Of the 12 unvaccinated participants who were eligible to get a COVID-19 vaccine, 9 (75%) decided to get the vaccine and secured a vaccine referral through *Mothers x Mothers*. Most gatherings had 5–7 participants, and no single gathering yielded more than 2 new vaccine referrals.

**Table 2:** Data for COVID-19-focused meetings of *Mothers x Mothers.*

Vaccination status before program	Obtained new COVID-19 vaccine referral through program				Total	
	Yes		No			
	N	%	N	%	N	%
Vaccinated	0	0	36	100	36	100
Unknown	0	0	1	100	1	100
Unvaccinated	9	75	3	25	12	100
Total*	9	18	40	82	49	100

*This data set excludes the first Mothers x Mothers gathering, a trial virtual gathering where some unregistered and uncounted participants joined through a shared Zoom link. From this point forward, RWN required pre-registration and put an attendance cap on gatherings to facilitate peer-to-peer discussion and post-event follow-up.

### 5.2 Building trust

RWN prioritized building and maintaining community trust. Community Health Promoters therefore took a respectful, linguistically and culturally responsive approach to conversations about the COVID-19 vaccine. RWN’s long-term goal with *Mothers x Mothers* was to gain trust so that community members would come back to RWN in the future for COVID-19 boosters, flu shots, pediatric vaccines, and other health resources. As one Community Health Promoter explained, “Once you gain [the mothers’] trust, it’s easier for them to bring their family on board, it’s easier for them to bring their friends on board, their other relatives… it spreads like wildfire.” The topic of trust came up several times in every interview conducted for this case study.

At the conclusion of each gathering, RWN collected feedback from *Mothers x Mothers* participants, either in person or via a phone call; Community Health Promoters helped by interpreting. RWN decided to ask relatively few questions at first, recognizing that questioning itself can become a barrier to participation. RWN’s original questions were:

1. Did your mind get changed about the vaccine?
2. Did you learn anything new about the vaccine?
3. In the future, would you bring friends? / Do you feel comfortable enough to bring friends?
4. What do you specifically want to learn about at the next event?

As RWN got more feedback from mothers and built more rapport, the questions were adjusted and broadened, thereby gathering needed information while maintaining an emphasis on participants’ levels of comfort and trust. Later questions included:

1. Were you able to speak honestly and ask questions you had during the *Mothers x Mothers* gathering? Did anything make you uncomfortable or nervous to share?
2. Are you COVID-19 vaccinated, including the booster?
3. Are you/your children HPV vaccinated?
4. Do you need a Health Promoter to set you or your child up with an appointment for the COVID-19 booster, flu shot, or HPV vaccine?
5. What health-related topic would you like to learn about next?

RWN evaluated how effectively it built trust by weighing (1) feedback collected from mothers after gatherings and (2) Community Health Promoters’ observations of how the groups interacted during gatherings. Reflecting on *Mothers x Mothers* as a whole, one Community Health Promoter said:

“Everyone who attends has said how much they enjoy their time coming to the mothers’ program, how well it has been run and how comfortable they feel sharing their thoughts in a judgment-free space.”

Another reflected on a series of gatherings as follows:

“I feel like it's honestly working, because I have people who aren't vaccinated still show up to every event, every time, never miss an event, and I'm like, ‘Okay, you're here for a reason’… so I'm going to keep doing what I'm doing.”

Finally, a Community Health Promoter observed after a typical gathering: “We had such a positive reception [when] we held it… we successfully held a safe space and we gave them information that they trusted.” Comments like these speak to RWN’s emphasis on trust as an integral ingredient of *Mothers x Mothers.*

### 5.3 Building capacity

RWN encouraged the Community Health Promoters to develop as community leaders so that, whether or not they continued with RWN, they could attain a level of confidence, connection, and empowerment that would allow them to play key roles around health in their communities. To evaluate the professional development of the Community Health Promoters, RWN conducted pre- and post-practice quality improvement assessments. These assessments gauged levels of self-confidence, ability to speak about the vaccine, and capacity to connect with community members and community leaders. Overall, RWN found that the Community Health Promoters approached their training and work with great care and dedication and developed accordingly. One Community Health Promoter spoke positively of her own growth, saying:

“When I started to do it, I was very scared… but when I came here they said, ‘No problem, you can learn.’ For me it was a good opportunity, health promotion…. I feel good because I’m doing something good for my community.”

Another said,

“I plan on going to medical school next year but I'm just like, I don't want to leave this program. This program has made such an impact. And it reminds me of programs we had back in the day in Clarkston that actually sponsored my family and brought them here.”

## 6 Discussion

### 6.1 Elevating community health promoters

RWN’s Community Health Promoters were involved in the *Mothers x Mothers* initiative in several notable ways. First, the Community Health Promoters were recruited and trained by RWN, a local community-based organization. Their training covered topics that might be relevant to CHWs anywhere, but with a special emphasis on serving refugee and immigrant women from local communities. Second, Community Health Promoters were involved in every phase of the initiative, from design to implementation to evaluation. This not only led to an initiative that was well suited for Clarkston refugees and immigrants, it also increased the engagement and interest of the Community Health Promoters–which in turn increased the engagement and interest of participating mothers. Third, the Community Health Promoters’ professional development was specifically included among the initiative’s goals. As part of its mission to empower refugee and immigrant women, RWN deliberately hired and trained women from local refugee and immigrant communities, and supported their growth.

### 6.2 Applying human-centered design principles

RWN, in close collaboration with IDEO.org, applied core aspects of human-centered design to plan an intervention for building vaccine awareness, confidence, and uptake among local refugee and immigrant mothers. The *Mothers x Mothers* initiative included strategies to provide information aligned with community values, engagement of peers as trusted messengers, and a commitment to affirm participants’ choices. The key elements of the human-centered design process were facilitated listening, ideation (i.e., creatively imagining solutions), prototyping (i.e., experimenting with proposed solutions), and implementation of the initiative (see Figure 1). RWN’s work serves as a foundational case study for increasing the evidence base around human-centered design as a critical tool in planning public health interventions, and the *Mothers x Mothers* toolkit has now been piloted across a handful of select sites. For other organizations considering implementing *Mothers x Mothers*, the toolkit is free to use and customizable (23).

### 6.3 Investing in peer support as an intervention model

When designing and implementing *Mothers x Mothers,* RWN and IDEO.org made a special effort to acknowledge and support the agency and experience of participants, allowing enough time for relationships between RWN and community members to develop and flourish. This approach aligns with Harris et al.’s and Schleiff et al.’s reviews of community-based peer support programs, which suggest that these programs are most effective when organizations share governance, respect cultural perspectives, and devote sufficient time and effort to assessing community needs and developing relationships (28, 35). Similarly, RWN and IDEO.org’s choice to focus on mothers as part of a campaign to address COVID-19 vaccine hesitancy squares with Schleiff et al.’s observation that “participatory women’s groups have been particularly successful in changing complex sets of behaviors that are embedded in cultural belief systems” (35).

Though *Mothers x Mothers* was admittedly a small-scale intervention for a specific demographic segment, it is worth briefly considering its possible ripple effects. When we take into account the role of these mothers in health decisions for their households and recall the high proportion of high-density households in Clarkston, we realize the possible exponential impact of the mothers’ improved vaccine confidence on vaccine uptake in their homes and communities.

## 7 Methodological constraints and strengths

This intervention and community case study have constraints and strengths worth noting. First, the design and implementation of *Mothers x Mothers* were tailored specifically to refugee and immigrant communities, which affects transferability and generalizability. Rather than viewing this as a limitation, however, we prefer to highlight that this intervention met the needs of local women from traditionally underserved populations. Second, recruitment for *Mothers x Mothers* involved self-selection and therefore our evaluation may reflect positive deviance. Third, our case study considered in detail the perspectives of *Mothers x Mothers* designers and group facilitators, without an equally in-depth look at the first-hand perspectives of participants. This limits our ability to draw definitive conclusions about why participants went to gatherings and how they changed their minds about the COVID-19 vaccine. Nevertheless, we were able to provide a rich description of *Mothers x Mothers’* program design and its key elements, which we hope will allow other community organizations to attempt similar approaches to planning and intervention.

## 8 Conclusion

In this case study we examine and describe features of the design, implementation, and evaluation of *Mothers x Mothers* that resulted in an increase in COVID-19 vaccine confidence and uptake among refugee and immigrant women in Clarkston, Georgia. When RWN launched the *Mothers x Mothers* initiative in late 2021, the participating mothers had been eligible for COVID-19 vaccination since March 2021 in the state of Georgia, yet many had not been vaccinated (36). Gatherings leveraged the influence and support of community peers, including CHWs, to shift attitudes and prompt action among unvaccinated participants—even in a climate of COVID misinformation and information fatigue.

Our goal in presenting this community case study is both to provide programmatic guidance and recommendations for other community organizations considering similar initiatives, and to increase the evidence base supporting small-scale community-led public health interventions. The *Mothers x Mothers* initiative succeeded in increasing vaccine acceptance, but the program was successful in two other respects that other organizations might seek to emulate as well. First, it empowered participating mothers to make informed health decisions. Mothers—described by RWN and IDEO.org as the “brokers,” “advocates,” and “gatekeepers” of their children’s and family’s health—play a significant role in community health decisions and behavior, so public health interventions that focus on mothers often have a broader impact. Second, the program built enough community trust to make future interventions possible, as evidenced by the continuing *Mothers x Mothers* gatherings that focus on health topics beyond COVID-19. Organizations designing similar programs might also seek to balance “success” here and now with the kind of long-term relationship-building that makes “success” more likely in the future.

## Data Availability

The original contributions presented in the study are included in the article/supplementary material, further inquiries can be directed to the corresponding author.
